# Speaking rate normalization with and without spatial segregation of simultaneous context sentences

**DOI:** 10.1121/10.0042965

**Published:** 2026-03-06

**Authors:** Dawson Stephens, Christian E. Stilp

**Affiliations:** 1 Department of Psychological and Brain Sciences, University of Louisville, Louisville, Kentucky 40292, USA; 2 Department of Speech Pathology and Audiology, Marquette University, Milwaukee, Wisconsin 53233, USA

## Abstract

Fast-rate speech can encourage perception of subsequent speech as longer-duration (e.g., longer voice onset time) and vice versa. Bosker, Sjerps, and Reinisch [Sci. Rep. **10**(1), 5607 (2020)] suggested that these temporal contrast effects (TCEs) (also known as speaking rate normalization) were immune to selective attention (to one of two simultaneous talkers). However, dichotic presentation of different talkers facilitated perception. Here, trials presented the same talker throughout speaking one sentence diotically, two simultaneous sentences diotically, or those two sentences dichotically before target words. TCE magnitudes were similar across all conditions, which suggests that spatial separation of simultaneous sentences does not shape TCEs.

## 1. Introduction

Acoustic properties of earlier sounds influence perception of later sounds. The present study examines speaking rate normalization, also known as temporal contrast effects (TCEs). Consider the example where listeners are categorizing a target word as “deer” or “tier” with stimuli that vary in voice onset time (VOT). When a preceding context sentence is spoken at a faster rate, the following target word is perceived as the longer-VOT “tier” more often; also, slower context sentences elicit more short-VOT “deer” percepts (e.g., Port, 1979; Diehl *et al.*, 1980; Summerfield, 1981; Kidd, 1989; Newman and Sawusch, 2009).

There is general consensus that amplitude modulations in speech play an important role in driving speaking rate normalization (Bosker and Ghitza, 2018; Oganian and Chang, 2019; King *et al.*, 2024). While accounts differ as to the fine details (see Discussion), they agree that the slow or fast arrival of amplitude modulations (delivered by syllables in speech) lead to the resulting perceptual shifts. Recent investigations have also considered contributions of top–down influences to this process. Bosker *et al.* (2020) presented listeners with context sentences spoken by two different talkers, one presented per ear (as in Newman and Sawusch, 2009), before the diotically presented target word. Listeners were instructed to attend to one of the two talkers; yet that instruction did not appear to matter. When both talkers spoke at the same rate, results mirrored those when listening to a single talker; when one spoke at a fast rate and the other at a slow rate, context effects were abolished. From these data, Bosker and colleagues concluded that speaking rate normalization is immune to selective attention. However, those stimulus arrangements did not challenge listeners' ability to segregate simultaneous talkers. In Newman and Sawusch (2009) and Bosker *et al.* (2020), all multi-talker conditions presented speech from two different adult talkers, one presented to each ear. The two talkers' voices were spatially separated as well as acoustically different from one another; both of these features play significant roles in segregating simultaneous sound sources (e.g., Kidd *et al.*, 1998; Freyman *et al.*, 1999; Brungart, 2001).

The present study examined how listeners' ability to segregate simultaneous speech affects TCEs in speech perception. In this study, all materials were spoken by a single talker. Trials presenting a single context sentence were predicted to produce the largest TCEs owing to their clear amplitude modulation structure. Trials presenting two context sentences simultaneously were predicted to produce smaller TCEs owing to mutual interference between their amplitude modulation structures. In these circumstances, sentences that could be spatially segregated (dichotic presentation) were predicted to produce less mutual interference and comparatively larger TCEs than when sentences were not spatially separated (diotic presentation). Thus, while all conditions are predicted to produce TCEs, their relative magnitudes were predicted to differ (single sentence > dichotic sentences > diotic sentences).

## 2. Methods

### 2.1 Participants

Forty-two undergraduate students participated in this study (20 with stimulus set 1; 22 with stimulus set 2). These sample sizes match those of recent studies of speaking rate normalization (Kahloon *et al.*, 2023; King *et al.*, 2024). All self-reported as being native English speakers with normal hearing. They participated in exchange for course credit.

### 2.2 Stimuli

Four context sentences from the Texas Instruments - Massachusetts Institute of Technology database (Garofolo *et al.*, 1990) were recorded by the second author. Sentences were organized into two stimulus sets of two sentences apiece, which were tested separately to assess generalizability and replicability of results. In the first stimulus set, sentences were “Upgrade your status to reflect your wealth.” (sentence 1) and “What did you mean by that rattlesnake gag?” (sentence 2). Both sentences had ten syllables and equal durations of 2098 ms. Speaking rates were manipulated in Praat (Boersma and Weenink, 2024) using the time-domain pitch synchronous overlap and add (TD-PSOLA) algorithm (Moulines and Charpentier, 1990). Fast versions of these sentences were produced by multiplying their durations by 50% (new fast durations = 1049 ms; speaking rate = 9.53 syllables/s). Likewise, slow versions of these sentences were produced by multiplying their durations by 150% (new slow durations = 3147 ms; speaking rate = 3.18 syllables/s).

In the second stimulus set, longer sentences with more syllables were selected. Sentences were “The misprint provoked an immediate disclaimer” (sentence 3) and “Even I occasionally get the Monday blues” (sentence 4). Both sentences had 13 syllables and equal durations of 2206 ms. As above, the speaking rates were manipulated using the TD-PSOLA algorithm in Praat. Fast versions of these sentences were produced by multiplying their durations by 50% (new fast durations = 1103 ms; speaking rate = 11.79 syllables/s). Likewise, slow versions of these sentences were produced by multiplying their durations by 150% (new slow durations = 3308 ms; speaking rate = 3.93 syllables/s).

Target words were the same “deer”–“tier” continuum used successfully in previous studies of speaking rate normalization (Kahloon *et al.*, 2023; King *et al.*, 2024). The second author was recorded saying the word “deer.” Using synthesis methods outlined by Winn (2020), a ten-step series of target words was created in Praat. This series perceptually varied from “deer” to “tier” by linearly increasing VOT from 21 ms at the “deer” endpoint to 82 ms at the “tier” endpoint. Secondary acoustic variations across the continuum from “deer” endpoint to “tier” endpoint included total duration (lengthening from 488 to 512 ms) and overall intensity (overall decrease of 1.4 dB).

Experimental trials were created by concatenating a target word (always presented diotically) to either a slow-rate context sentence (or sentences) or a fast-rate context sentence (or sentences), separated by a silent 50 ms interstimulus interval.

### 2.3 Procedure

After providing informed consent, the participant was seated in a sound-attenuating booth (Acoustic Systems, Inc., Austin, TX). Stimuli were digital-to-analog converted by RME HDSPe AIO sound cards (Audio AG, Haimhausen, Germany) on personal computers and passed through a programmable attenuator (TDT PA4, Tucker-Davis Technologies, Alachua, FL). Stimuli were presented over circumaural headphones (Beyerdynamic DT-150, Beyerdynamic Inc., Farmingdale, NY) at a mean presentation level of 70 dB sound pressure level. A custom script in matlab (Mathworks Inc., Natick, MA, USA) led the listener through the experiment, which was self-paced. Participants received a single set of general instructions, asking them to listen carefully to the series of sounds or sentence ending in the target word on each trial. There were no directions to attend to a specific sentence or ear.

Participants first completed a practice block of 20 trials. Each trial presented a medium-rate context sentence from the AzBio corpus (Spahr *et al.*, 2012) spoken by the second author, followed by one of the two endpoints words of the “deer”–“tier” continuum. At the end of each trial, a response window appeared with buttons labeled “deer” (top button) and “tier” (bottom button). Participants used the mouse to click the button corresponding to their response in two-alternative forced choice format. Feedback was provided on each trial. All participants met the performance criterion of 80% correct that was required to continue to the main experiment.

The main experiment consisted of four blocks: two blocks presenting a single sentence on each trial (sentence 1 or sentence 2 in stimulus set 1; sentence 3 or sentence 4 in stimulus set 2) and two blocks presenting two simultaneous sentences on each trial (diotic sentences or dichotic sentences: Sentence 1 and sentence 2 in stimulus set 1; sentence 3 and sentence 4 in stimulus set 2). Each block had 160 trials: 80 trials presenting slow-rate sentences and 80 trials presenting fast-rate sentences. In the dichotic sentences block, speaking rates and sentences were heard an equal number of times in each ear. Stimuli were presented in random order within a block, and the blocks were tested in counterbalanced orders. No feedback was provided. Participants took small breaks between blocks if they chose. The entire experiment took approximately 50 min.

## 3. Results

Data were analyzed via generalized linear mixed-effects modeling in *R* (R Core Team, 2025) using the lme4 package (Bates *et al.*, 2014). The dependent variable was participants' responses modeled as a binary variable (0 = “deer,” 1 = “tier”). Responses were predicted based on the fixed effects of target (continuum steps numbered 1–10 them mean-centered), condition (four-level factor with dichotic presentation as the default), speaking rate (sum-coded, with slow coded as −0.5 and fast coded as +0.5), and all interactions between these fixed effects. Random intercepts for listeners were entered into the model, then random slopes were added and tested iteratively via χ^2^ goodness of fit tests. Random slopes that resulted in significant improvements in model fit were retained; others that did not improve model fit were rejected. The final model for analyzing the first stimulus set included random slopes for target and speaking rate, and the final model for analyzing the second stimulus set included those terms as well as random slopes for condition.

**Fig. 1.**
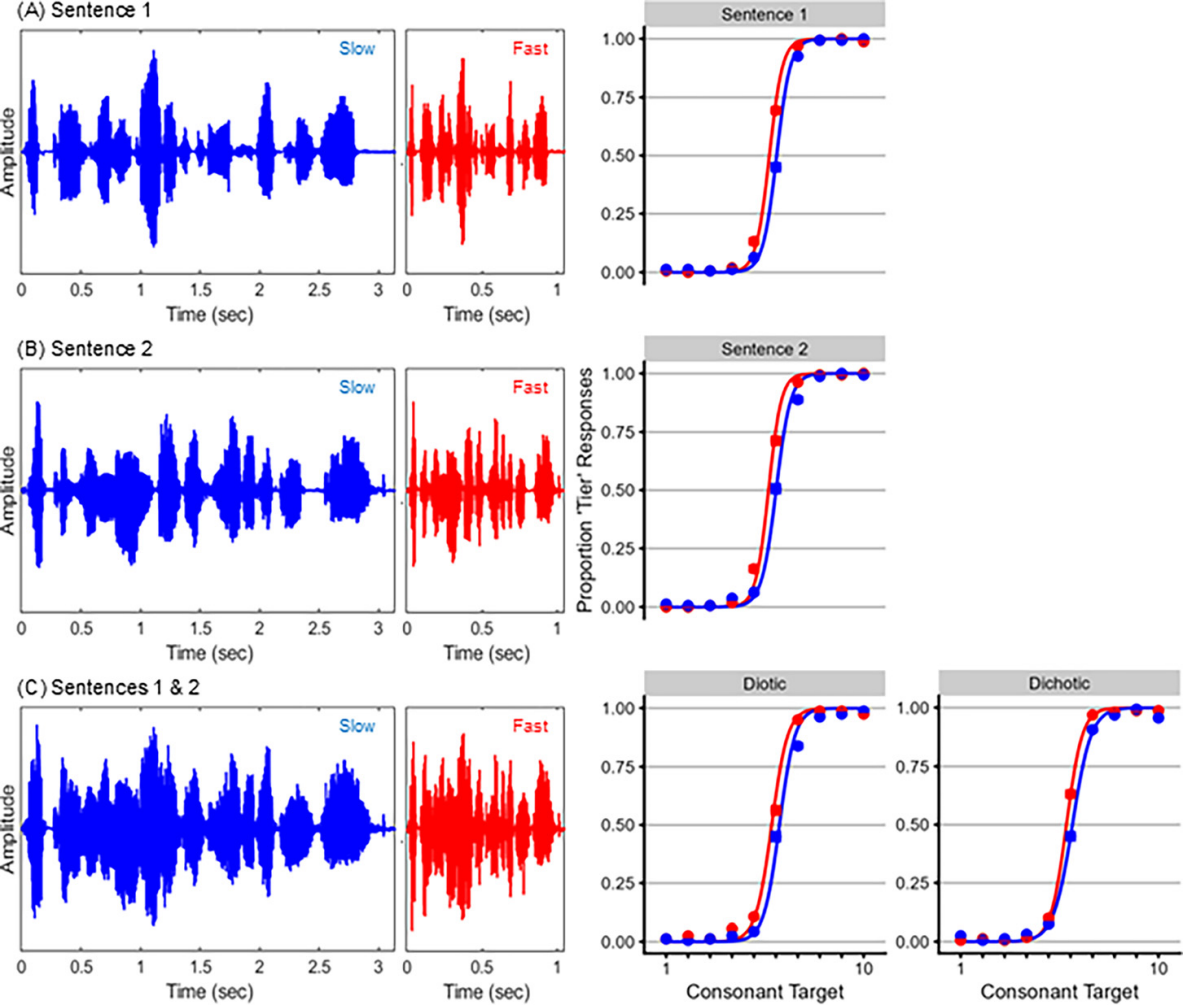
Stimuli and results from stimulus set 1. (A) Waveforms for the slow-rate (left, in blue) and fast-rate (center, in red) context sentence 1, “Upgrade your status to reflect your wealth.” Behavioral results are shown at right, with the proportion of “tier” responses plotted as a function of word in the target continuum. Circles indicate mean proportion of “tier” responses to each target word, and curves are the fitted psychometric functions from the mixed-effects regression analyses. (B) Waveforms and behavioral results for context sentence 2, “What did you mean by that rattlesnake gag?”. (C) Waveforms and behavioral results when context sentences 1 and 2 were presented simultaneously. The left set of behavioral results correspond to when sentences were presented diotically; the right set of behavioral results correspond to when sentences were presented dichotically.

**Fig. 2.**
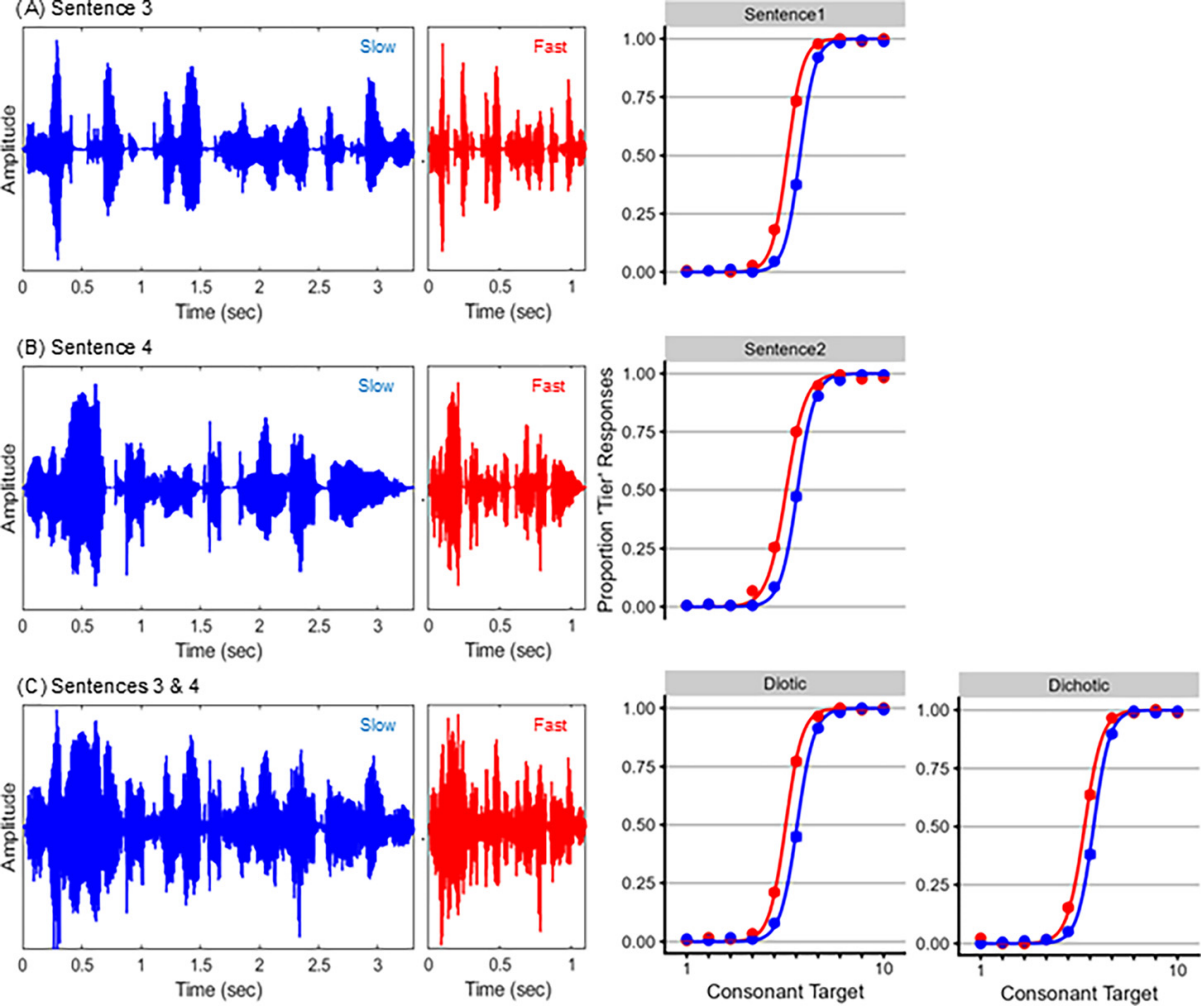
Stimuli and results from stimulus set 2. (A) Waveforms for the slow-rate (left, in blue) and fast-rate (center, in red) context sentence 3, “The misprint provoked an immediate disclaimer.” Behavioral results are shown at right, with the proportion of “tier” responses plotted as a function of word in the target continuum. Circles indicate mean proportion of “tier” responses to each target word, and curves are the fitted psychometric functions from the mixed-effects regression analyses. (B) Waveforms and behavioral results for context sentence 4, “Even I occasionally get the Monday blues.” (C) Waveforms and behavioral results when context sentences 3 and 4 were presented simultaneously. The left set of behavioral results correspond to when sentences were presented diotically; the right set of behavioral results correspond to when sentences were presented dichotically.

See the supplementary material for full results from all mixed-effect regression analyses. For the first stimulus set, each condition produced a speaking rate normalization effect (fixed effects of speaking rate: all *Z* > 2.23, *p* < 0.05; Fig. 1). Contrary to predictions, speaking rates did not differ between any conditions (rate by condition interactions: all *Z* < 1.51, *p* > 0.19). For the second stimulus set, each condition again produced a speaking rate normalization effect (fixed effects of speaking rate: all *Z* > 5.71, *p* < 0.0001; Fig. 2). Again, contrary to predictions, speaking rates did not differ between any conditions (rate by condition interactions: all *Z* < 1.52, *p* > 0.13).

## 4. Discussion

Speaking rate normalization (TCEs) has long been studied by presenting a single context sentence before the target word on each trial. While this has facilitated bottom–up approaches to speaking rate normalization that focus on stimulus acoustics (such as amplitude modulation rate), contributions of top–down influences to this process remained unclear. Bosker *et al.* (2020) concluded that selective attention plays no part in speaking rate normalization, but the simultaneous talkers differed from each other both acoustically and spatially (see also Newman and Sawusch, 2009). This might have helped listeners separate the talkers, so that the amplitude modulation structures of their speech could still effectively produce TCEs. Here, acoustic differences were controlled (by using one talker) and spatial separation contrasted to vary the integrity of amplitude modulation structure. TCEs were predicted to be largest when hearing a single context sentence (diotic presentation), followed by simultaneous context sentences that were spatially segregated (dichotic presentation of different sentences), and the smallest TCEs when sentences were not spatially separated (diotic presentation of combined simultaneous sentences). The present results did not confirm these predictions, as observed in two different stimulus sets.

Key differences across the present experimental paradigm and that of Bosker *et al.* (2020) must be noted. In Bosker *et al.* (2020), speech from different talkers was presented, sometimes speaking at the same rate as each other and sometimes at opposing rates. Here, the same talker was always presented, and simultaneous sentences were spoken at the same rate on each trial. Bosker *et al.* (2020) utilized an attentional manipulation, instructing listeners to detect keywords spoken by one of the two simultaneous talkers on each trial; no such attentional manipulation was utilized here. Also, there are claims that lexical rate effects (involving detection of syllables, as tested by Bosker *et al.*, 2020) qualitatively differ from rate effects at the level of the speech sound (see Baese-Berk *et al.*, 2019 for discussion). Nevertheless, results from Bosker *et al.* (2020) were explained parsimoniously by stimulus acoustics despite relatively easy (acoustic and spatial) stimulus segregation without any effects of their attentional manipulation (see also Bosker *et al.*, 2017, where attentional manipulations via cognitive load did not affect the magnitudes of acoustic context effects). Here, results were consistent with stimulus acoustics irrespective of spatial segregation of simultaneous sentences from a single talker.

Some uncertainty remains regarding the exact nature of the bottom–up information driving TCEs. While general consensus exists about amplitude modulations in the preceding acoustic context playing a key role, accounts differ as to which aspect of these modulations is responsible. Bosker and Ghitza (2018) proposed that speaking rate normalization is the product of cortical neurons in the theta frequency range (3–9 Hz) entraining to amplitude modulations in speech. This entrainment is sustained into presentation of the target, which results in cortical oversampling (for entrainment to fast-rate speech) or undersampling (for entrainment to slow-rate speech) of the speech signal, resulting in the perceptual shift (TCE). Conversely, Oganian and Chang (2019) and Oganian *et al.* (2023) proposed that it is not the peaks of modulations (e.g., relative maximum in amplitude at the center of a consonant-vowel-consonant syllable) that drive this perceptual normalization but their rapid onsets (the “acoustic edges”). When speaking rates were slowed to one-third or one-quarter of their native rates (as to better separate the amplitude modulation onsets from their centers/peaks), electrocorticography and magnetoencephalography responses in the delta (roughly 1–4 Hz) and theta ranges (roughly 4–8 Hz) better correlated with these acoustic edges than envelope peaks. Further, neural encoding of the magnitudes of acoustic edges was invariant to sentence speaking rate. Oganian *et al.* (2023) offered this automatic speech rate normalization as a mechanism that could underlie TCEs. However, these methods did not measure acoustic edges in fast-rate speech, as increasing speaking rate would bring acoustic edges and moments of peak amplitude closer together in time, making it very difficult to discern which acoustic landmark was responsible for a given evoked neural response. The debate between these oscillatory vs evoked accounts is ongoing (e.g., Duecker *et al.*, 2024; Damsma *et al.*, 2025). Future research that isolates the contributions of these candidate accounts to speaking rate normalization will be highly revealing.

This study had several limitations. First, presenting two simultaneous context sentences (as Bosker *et al.*, 2020 did) was predicted to compromise the amplitude modulation structure of speech rate. However, it did not eliminate cues to speech rate, as amplitude modulations were still present in the stimuli [cf. Figs. 1(C) and 2(C)]. Future research may test this prediction more strongly by using context stimuli with weaker modulation cues (e.g., multi-talker babble). Second, each experiment used a small number of sentences to limit overall acoustic variability, which has been shown to diminish TCE magnitudes (King *et al.*, 2024). However, repeating a small number of sentences could promote familiarity, which would then aid in perceptually separating simultaneously presented sentences. Future research might pursue a balance between the acoustic variability and familiarity of context sentences. Third, sentence duration covaried with speaking rate (slow-rate sentences had longer durations than fast-rate sentences). While listeners could have used overall duration as a rate cue, TCEs still occur when context duration is fixed (Bosker, 2017), suggesting that speaking rate normalization does not wholly rely on overall duration information. Finally, sample sizes were chosen to be comparable to those of related studies of TCEs (Bosker *et al.*, 2020; Kahloon *et al.*, 2023; King *et al.*, 2024). While they might have benefited from more statistical power, replicating the null differences in TCEs across conditions in two stimulus sets helps support the conclusions drawn here.

Acoustic context effects have a long history of being studied using stimuli presented in quiet (but see Newman and Sawusch, 2009; Stilp, 2017; Bosker *et al.*, 2020; Reinisch and Bosker, 2022 for competing-speech and background noise manipulations). Everyday listening often requires the listener to perceive speech amidst background noise. This raises important questions about how background noise affects the amplitude modulations thought to drive TCEs. Amidst a competing sound source, amplitude modulations in the target speech could be masked or reduced through modulation interference. Conversely, an amplitude modulation in the competing sound source might align with silence in the target speech (thereby increasing the overall modulation rate) or with another modulation in the target speech (increasing its modulation depth). Any or all of these scenarios would be expected to alter evoked neural responses to the incoming speech (whether to the modulation peaks and/or to the acoustic edges) with potential behavioral consequences as well. While results here did not differ across one-sentence and two-simultaneous-sentence conditions, this does not rule out perceptual sensitivity to these aspects of speech perception amidst competing sound sources. Further research in this direction will illuminate the degrees to which these acoustic context effects shape everyday speech perception.

## Supplementary Material

See the supplementary material for full results from all mixed-effect regression analyses.

## Data Availability

All stimuli, data, analysis scripts, and supplementary analyses are available at https://osf.io/8jsa5/.
